# Maternal Employment and Child Malnutrition in Ecuador

**DOI:** 10.3390/ijerph20136253

**Published:** 2023-06-29

**Authors:** José Andrade, Joan Gil

**Affiliations:** 1 Max Planck Institute for Demographic Research, Konrad-Zuse-Str., 18057 Rostock, Germany; andrade@demogr.mpg.de; 2 Facultat d’Economia i Empresa, Universitat de Barcelona (UB), Diagonal Ave. 696, 08034 Barcelona, Spain; 3 Barcelona Economic Analysis Team (BEAT), Universitat de Barcelona (UB), 08034 Barcelona, Spain

**Keywords:** maternal employment, stunting, wasting, underweight, overweight

## Abstract

Background: This paper estimates the causal impact of maternal employment on childhood malnutrition status in Ecuador to understand the trade-off between the time mothers devote to work and the time they dedicate to child-caring activities. Methods: We use the instrumental variables (IV) approach and exogenous cantonal variation in maternal labor market conditions to account for the potential endogeneity of mothers’ employment. The analysis employs the Ecuadorian National Health and Nutrition Survey 2018 and the Living Conditions Survey 2014. Results: The IV estimations indicate that maternal employment increases the probability of having stunted children by between 4.2 and 18.1 percent, while no significant effect is found in the case of children suffering from wasting, being underweight, or being overweight. The effect of maternal employment on stunting is stronger among mothers with high education and living in high-income households. Inconclusive effects of mothers’ overweight status are reported. The results are robust to several robustness checks. Conclusions: Overall, our findings suggest that the additional income that a working mother may obtain (the income effect) does not offset the loss of time available for direct childcare (the time constraint) in terms of child health status, and this effect is even more apparent for more affluent and more educated mothers. Government interventions, including effective conditional cash transfers and/or in-kind family policies, intended to reduce the cost of raising children among vulnerable families appear to be aligned with our findings.

## 1. Introduction

Over the last three decades, child malnutrition in its various forms has become one of the most significant health problems in low- and middle-income countries, and Ecuador is no exception [1]. The impact that malnutrition can have over an individual’s lifetime needs to be better understood. For instance, stunting in childhood is related to infections, developmental deficits, brain damage, adult non-communicable diseases, early mortality, poorer school performance, and lower productivity and income as an adult [2,3,4]. Additionally, children born to stunted adults are more likely to suffer stunting themselves [5].

Although public healthcare expenditure has increased dramatically in Ecuador over the last decade [6], indicators of child malnutrition have not improved as expected. For instance, the Ecuadorian National Statistical Office reported that 25% of children under five (U5) years old were stunted (children considered too short for their age) in 2012. This figure fell only slightly to 23% in 2018. Similar figures have been reported for wasting (children considered too thin for their height) and being underweight [1,7], while the percentage of overweight U5-year-old children increased in the same period. Among Latin American countries, Ecuador has the highest prevalence of stunting after Guatemala [8], with rates comparable to sub-Saharan countries [9].

Policy responses to tackle malnutrition in Ecuador have not succeeded in reducing this health issue significantly. Government interventions such as the Programa Aliméntate Ecuador [10], which includes the provision of micronutrients such as iron, zinc, vitamin A, and folic acid, have not managed to reduce malnutrition among U5-year-old children. Rivera [11] claims that the reasons for this lack of success can be related to insufficient institutional coordination (nutrition and primary health programs, for example, operate separately), which has tended to undermine the efficient allocation of resources and management capacities between central and local governments in their efforts to address child malnutrition. In addition, the absence of government policy evaluations has restricted analyses of the success or failure of these policies, the degree of transparency in policy processes, and the efficient targeting of interventions.

In parallel, women’s participation in the labor market has increased remarkably over the last decade [12], which raises the question as to whether maternal employment is a detrimental factor in child health [13,14]. Mothers’ employment increases household income, which improves families’ wellbeing and, in turn, children’s nutritional and health status. However, mothers who work outside the home have less time for breastfeeding and childcare activities, which can be considered detrimental for their children’s nutrition and health outcomes. We implicitly assume that non-employed mothers devote their time to feeding and caring for their children. Therefore, it is crucial that in-depth studies be undertaken of the trade-off between mothers that generate additional income, on the one hand, and those that provide full-time child care, on the other, especially in low- and middle-income countries. Such analysis should help in understanding the net effect of maternal employment on children’s nutritional status, which, a priori, is not obvious, and in designing better-informed policy interventions. Yet, clearly, adverse socioeconomic conditions may not give mothers a choice as to whether to go out to work or to stay home and take care of their children. They may be forced to work to generate enough income to escape poverty. For such population groups, it is fundamental that we capture the effects of maternal labor supply on child nutrition.

This study aims to estimate the causal effect of maternal labor supply on children’s nutritional status (that is, stunting, underweight, wasting, and overweight) in Ecuador, considering the mothers’ wish to form part of the labor force or to remain economically inactive, and accounting for the potential endogeneity of maternal employment—that is, the presence of unobserved characteristics correlated with mothers’ decisions to work and with their children’s health and simultaneity issues—and the heterogeneous impacts on population subgroups. We estimate this causal effect by employing an instrumental variables (IV) approach, using the average employment rate among women and the average number of hours worked at the cantonal level as our instruments. The main IV findings suggest that the probability of having a stunted U5-year-old child is between 4.2 and 18.1 percent higher for employed mothers than it is for unemployed or inactive mothers. This estimate is large and significant. No evidence of the impact of maternal employment on wasting, underweight, or overweight is found. Moreover, the effect size of maternal employment appears to be driven by moderately stunted children. Interestingly, we show that the effect of maternal employment on child malnutrition is greater among mothers with high education and living in high-income households. This points to the dominance of the time constraint over the income effect. The IV results indicate that, in contrast to the ordinary least squares (OLS) strategy, maternal employment increases a child’s risk of experiencing stunting to a greater extent.

This paper contributes to the literature in several ways. First, our findings constitute some of the first evidence to quantify the causal effect of maternal labor supply on child malnutrition in South America. Second, the childhood outcomes herein are constructed by employing more reliable, directly measured anthropometric data that do not suffer from self-reported biases. Third, in contrast to Rashad and Sharaf [15], this paper uses area-level maternal labor market conditions as an instrument of maternal employment and explores heterogeneous effects among population subgroups.

The rest of the paper is structured as follows: Section 2 discusses empirical findings in the literature regarding the effects of the maternal labor supply on children’s health status. Section 3 describes in detail the empirical specification and methodology employed, while Section 4 outlines the characteristics of the data used. Section 5 presents the empirical results, and Section 6 reports some robustness checks. Finally, Section 7 offers a discussion of our findings and some concluding remarks.

## 2. Literature Review

The empirical literature on how parental labor supply affects child health status is mostly based on the child health production function [16,17,18]. Several studies have adopted this theoretical framework and focused on how parental labor supply affects child health indicators. For instance, Morrill [19] estimated the effects of maternal employment on the health of US school-age children measured as overnight hospitalizations, asthma episodes, injuries, and poisonings. Using an instrumental variable (IV) approach, where the instrument for maternal labor supply was the exogenous variation in each child’s youngest sibling’s eligibility for kindergarten, she found significant, high estimates suggesting that maternal employment increases the probability of a child having a negative health episode. Meyer [20] examined the impact of maternal employment on the risk of childhood overweight in Germany, considering the number of younger siblings in the household as an instrument. She showed that the probability of being overweight increased due to maternal full-time employment. Thus, she attributed unhealthy behavior in children, in terms of diet and activity, to the reduction in maternal time devoted to them. In a similar vein, Anderson et al. adopted an IV strategy and found that American mothers who work more hours per week are more likely to have an overweight child [21]. Similarly, Datar et al. investigated the relationship between a mother’s working hours, a child’s body mass index (BMI), and obesity in the US using an IV approach [22]. In this study, the instrument employed was maternal work based on state-level variations in labor market conditions. The authors found a positive association between maternal working hours and child BMI and obesity, particularly in households with higher socioeconomic levels. Interestingly, Cawley and Liu showed that maternal employment is associated with less time spent on grocery shopping and on cooking, eating, playing with, supervising, and caring for children, particularly among mothers with young children [23]. This sheds some light on the possible causes of childhood obesity when a mother is engaged in the labor market.

In contrast with previous findings, Bishop [24] used economic conditions in the mother’s area as instruments and sibling difference models and found that part-time and full-time maternal work decreased excess bodyweight among Australian youth. Interestingly, a higher income did not appear to be responsible for this effect. Mocan et al. studied the impact of maternal earnings, determined by hours of work and wages, on the birth weight and gestational age of infants in the US within an IV framework [25]. They concluded that labor earnings had a positive but small effect on the birth weight and gestational age of the newborns of low-skilled mothers, while an increase in the earnings of high-skilled mothers did not have any effect on the health of newborns. Finally, Greve [26] found no effect of maternal working hours on child overweight status in Denmark, in contradiction with studies from other countries.

However, there is a scarcity of studies of the causal impact of maternal employment on children’s nutritional status measured by anthropometric indicators (i.e., stunting, wasting, underweight, and overweight). Existing studies have mainly been carried out in developing countries and use older samples of children and/or youth. The broader correlational literature on the association between maternal employment and child malnutrition status in developing countries is generally inconclusive. For instance, mothers’ labor supply was reported as being positively related to child malnutrition in some Asian countries and large cities in India [27,28]. In contrast, a study conducted in Panama found that maternal employment was not correlated with child nutritional status [29]. For an extensive review, see Glick [30]. One of the most similar studies to the one conducted herein is that of Rashad and Sharaf [15], who investigated the causal impact of maternal employment on malnutrition indicators among U5-year-old children in Egypt, using local employment conditions as their instrument to account for the endogeneity of maternal employment. In short, the current body of evidence points to a strong positive causal effect of maternal employment on the probability of stunting and wasting in children.

## 3. Empirical Specification and Methodology

### 3.1. Child Health Production Function

The theoretical framework of this paper is based on the child health production function, whose foundations lie in the theory of the allocation of time between work and the production of household goods [16]. Households are both consumers and producers. In line with microeconomic theory, they produce goods by combining inputs and time. Jacobson [18] extended Grossman’s [17] model by proposing a model in which the family is the producer of health. In this sense, each family member will produce their own health and that of other family members’ by investing resources in health until the rate of marginal consumption benefits equals the rate of marginal net effective costs of health capital.

Several papers have used a child health production function accounting for endogeneity when estimating the relationship between parental labor supply and child health [24,26,30,31]. In this setting, households invest time and goods (inputs) to produce child health (output) and maximize their utility by allocating parental time and income to produce time-intensive activities, such as devoting quality time to their children, preparing healthy, nutritious food, and providing medical care. Since time and income are scarce commodities, two effects emerge when parents, particularly mothers, face the decision of going out to work. If mothers reduce the time invested in taking care of their children, this can be detrimental to the child’s health capital stock. However, mothers who devote greater time to work can earn an income and purchase more and better-quality health inputs, which increases child health output. The trade-off between these two effects leads to a theoretically ambiguous net effect [24,31].

### 3.2. Econometric Model

We estimated the effect of maternal labor status on children’s health outcomes according to the following structural equation:

(1) $$ChildH_{ij}=\alpha +\beta MLS_{ij}+\gamma X_{ij}+\varepsilon_{ij}$$

where $ChildH_{ij}$ represents the health outcomes of interest to child *i* of mother *j*, i.e., stunting, wasting, underweight, and overweight. $MLS_{ij}$ is a vector of maternal labor supply variables, $X_{ij}$ is a vector of social, economic, and demographic characteristics of the child, mother, and his/her family, and $\varepsilon_{ij}$ is the error term of the equation. Notice that β in Equation (1), the parameter vector of interest captures the effect of maternal employment on child malnutrition. Estimating this model by OLS or binary response models would yield inconsistent, biased estimates that only capture a correlation between child health and maternal employment. The presence of omitted variables and simultaneity issues makes MLS a potentially endogenous regressor (cov(MLS, ε)≠0). This would produce inconsistent OLS estimates. Note that OLS estimates would appear downwardly biased if, for instance, employed mothers also had high skills in caring for children, which would act as a protective child health factor. In contrast, upwardly biased estimates would be observed if working mothers were less inclined to carry out childcare activities.

To address this problem, we used an instrumental variables (IV) framework on a two-stage least squares (2SLS) model [32]. The first stage estimates the endogenous variable MLS on a vector of exogenous instruments Z that do not directly affect children’s health and all the remaining exogenous covariates of the structural Equation (1). The validity of the instrument Z rests on two assumptions: (i) the relevance condition where the correlation between MLS and Z is different from zero [cov(Z,MLS)≠0]; (ii) the instrument and the error term in Equation (1) are uncorrelated, cov(Z,ε)=0. If these assumptions are satisfied, the instrument is considered “exogenous” in Equation (1).

(2) $$MLS_{ij}=\alpha_{1}+\beta_{1}Z_{ij}+\gamma_{1}X_{ij}+\mu_{ij}$$

The second stage is the OLS regression of Equation (1), which includes the prediction ($\hat{MLS}$) from the first stage estimation and serves as an instrument for the endogenous variable. Thus, the second stage equation is:

(3) $$ChildH_{ij}=\alpha +\beta_{IV}{\hat{MLS}}_{ij}+\gamma_{IV}X_{ij}+\omega_{ij}$$

In this case, if the aforementioned assumptions about the instrument hold, the parameter $\beta_{IV}$ is consistent and becomes the causal effect of maternal labor supply on child malnutrition status.

The estimation of linear regression models on dichotomic outcomes (=1 if the child experiences stunting, wasting, underweight, or overweight, and 0 otherwise) would be adequate as the sample size employed is moderately large and the mean of the outcome variables is centered [33]. However, as a robustness analysis, we ran the seemingly unrelated regression (SUR) bivariate probit model [34,35]. In this framework, structural and reduced form equations are estimated together based on a binary choice probit model, allowing the errors of both equations to be correlated (i.e., E(ε)=E(μ)=0,var(ε)=var(μ)=1, cov(ε,μ)=ρ). Note that if the parameter ρ is significantly different from zero, which indicates the existence of unobservable factors related to maternal employment and child malnutrition, then the bivariate probit will provide consistent estimates of the effect of maternal labor supply.

### 3.3. Endogeneity of Maternal Employment

Maternal labor supply is potentially endogenous when its association with child health is analyzed in Equation (1). As discussed above, there are two main reasons for this. The first is associated with the presence of unobserved characteristics correlated with maternal labor supply and child health outcomes. For instance, mothers with low interest/motivation, or skills/abilities may work less and take less care of their children than mothers with high interest/skills, and health knowledge [21,24,26]. Similarly, Morrill [19] argued that unobserved characteristics related to mothers’ preferences and skills might influence the choice of whether to work or not, which would lead to a selection bias in the sample of mothers.

More interestingly, Cavapozzi et al. argued that unobserved cultural or gender norms may affect female labor market behavior [36]. For example, a mother’s decision to work can be determined by the gender roles in her household. Similarly, a woman whose peers present gender-egalitarian behavior is more likely to work than a woman with more traditional peers. This gender role might also affect children’s nutritional status in terms of the time spent by mothers taking care of their children.

Further potential unobserved characteristics correlated with maternal employment and parental inputs might include the desire to continue a pregnancy (wantedness), the taste for risky behaviors, and maternal health endowment [37]. For instance, women who take care of themselves immediately after falling pregnant because they want to carry their pregnancy to term are more likely to eat nutritiously, avoid large amounts of stress and potentially harmful substances, and engage in appropriate physical exercise. As such, the child’s nutrition will be better due to want. In contrast, the taste for risky prenatal behavior (i.e., cigarette smoking, alcohol consumption, and illicit drug use) leads to inadequate parental care and consequently detrimental child health status. Similarly, maternal health endowment prior to and during pregnancy may affect the decision to work and impact the child’s nutritional status.

The second reason is related to a reverse causality problem that emerges when a mother chooses whether to work or not based on her child’s health status or decides to quit her job to care for a child that suffers detrimental health conditions, inducing a negative correlation between employment and child health [24]. Indeed, several studies have reported evidence of how child health status influences parental working decisions [38,39,40].

### 3.4. Instrumental Variables

This paper instrumented maternal labor supply (MLS) using the average employment rate and the average number of daily hours worked by employed and self-employed women measured at the cantonal level. These exogenous instruments were assumed to capture local labor market conditions and the labor demand for women. The combination of multiple IVs for a single endogenous regressor is often employed by researchers to boost statistical efficiency. In our case, this involves the use of two datasets (as highlighted in Section 4). Higher average employment rates and an intensive margin of maternal employment in a particular area make it easier for resident mothers to find a job. In contrast, high unemployment or shorter working times in an area are barriers to finding a job. Both instruments are external factors for mothers and households and are determined exogenously by local economic conditions [15,22,24].

Previous studies have used such instruments to deal with endogeneity problems in their efforts to find a causal relationship between mothers’ labor supply and child health status [15,21,22,24,26]. A total of 206 cantons (ENSANUT 2018) and 213 cantons (ECV 2014) in Ecuador were considered to compute the instruments. Reasonable variation was found between the geographical areas. Given the large number of cantons considered, disaggregation of the instruments by sector and/or industry was deemed inadequate due to the small number of observations.

Note that among self-employed women, only those working outside the home were included in the definition of maternal employment, to exclude mothers who earn an income and devote time to childcare at the same time. This avoids an in-built association. Another assumption would be to assume the absence of a trade-off if the volume of tasks for this group of women were to preclude them from dedicating time to their children. Moreover, the reference population for calculating the instrument was all working-age women (15+), including economically active (employed and unemployed) and inactive women. It was assumed that the chances of finding a job affect not only mothers who are already employed but also those who are looking for a job and those who have decided to study or stay at home, looking for new opportunities to enter the economically active population.

In addition, to guarantee the exogeneity of the instruments in the structural equation, other cantonal-level defined covariates such as poverty, income inequality (as measured by the Gini index), and mothers’ poor health status, which should be correlated with child health conditions, were included in the second stage (Equation (1)). Note that the cantonal-level covariates included in Equation (1) were compared to cantonal-level covariates estimated from the Labor Force Survey (Encuesta Nacional de Empleo, Desempleo y Subempleo, ENEMDU 2018) to guarantee their consistency. A comparison (Figure A2 in Appendix A) shows that these regional measures are highly similar in the two surveys. Thus, we addressed potential heterogeneity across cantons that might have confounded the IV estimates.

Yet, it is possible that mothers make location decisions based on unobserved factors also correlated with their children’s health status that ultimately affect the exogeneity of the instruments [24]. By using the emigration component of the ECV 2014, we found that around 77.9% of the mothers in our sample have not migrated within the country in the last 10 years (87.1% in the last 5 years). We understand that this rather low mobility rate may not greatly affect self-selection into cantons.

## 4. Data and Variables

This study used two sources of Ecuadorian cross-sectional microdata: the National Health and Nutrition Survey (ENSANUT) 2018 and the Life Conditions Survey (ECV) 2014, both produced by the National Institute of Statistics and Censuses (INEC). Two datasets were used because information on the number of hours worked by the interviewees was only present in the ECV survey, while both surveys contained parental employment status data. Fortunately, both datasets contained the information needed to run the econometric models, which seek to generate indicators on the main health situation and living standards of the Ecuadorian population [41,42]. The only exception is potable water control, which was not included in ECV 2014. Note that analyses were run separately on both datasets. The data requirements were extensive and included information on anthropometrics, children’s and women’s health information, parental socioeconomic variables, and other household information.

The sample design implemented in ENSANUT 2018 followed a two-stage probabilistic stratified sample. The first stage stratified the sample through primary sampling units (PSU), while the second stage considered a certain number of dwellings randomly (18 dwellings on average) per PSU [43]. Thus, the survey investigated a total of 2591 clusters and 43,311 dwellings. The total sample size was 168,747 individuals [1]. Specifically, the ENSANUT 2018 sample used in this study considered 17,587 mothers matched with 20,204 U5-year-old children. Similarly, ECV 2014 was produced under the same sample design as ENSANUT 2018 [41] and the working sample considered 8824 mothers matched with 10,837 children. Both surveys include canton identifiers so that regional market conditions can be estimated more precisely.

### 4.1. Child Nutritional Status

The key outcomes investigated to characterize child malnutrition status were three measures of undernutrition (stunting, wasting, and underweight) and overweight status. According to the World Health Organization (WHO), stunting (low height-for-age) is the result of chronic or recurrent undernutrition and is usually related to deprived socioeconomic status, poor maternal health and nutrition, and inappropriate feeding and care in early life [44]. Stunting appears to hold children back from reaching their physical and cognitive potential. Wasting (low weight-for-height) indicates recent and severe weight loss because of the scarcity of meals and/or the impact of infectious diseases. Children with a low weight-for-age are classified as underweight. In contrast, overweight status refers to a child who is too heavy for his or her height due to an excessive accumulation of fat, which impairs children’s health.

To assess the children’s nutritional status, we used *directly* measured anthropometric information available in both datasets. Each survey included the sex and age in days of children U5 years old, their weight in kilograms (kg), and their height/length in centimeters (cm). Using this information and the child growth standards developed by the WHO [45], we estimated the standard deviation (SD) score (Z-score), one of the most common and frequently used indexes in the literature [46,47]. Therefore, we used three Z-scores (continuous and normally distributed variables) to define the anthropometric indicators: height-for-age Z-score (HAZ), weight-for-age Z-score (WAZ), and weight-for-height Z-score (WHZ).

Following the WHO [48], a child with a HAZ, WHZ, or WAZ < −2 SD is considered stunted, wasted, or underweight, respectively, while an overweight status corresponds to a child with a WHZ > +2 SD. Moreover, this study followed WHO recommendations for dropping values that are outside the range for plausible Z-scores (WHO, 2006).

Note that the length/height, and weight values of U5 children were measured in survey data following a specific procedure [49]. Each child was measured twice using a weight scale and stadiometer. The medical device was reset each time. Children under the age of 2 were measured in the supine position using a specific stadiometer. A third measurement was taken when the difference between the first two measurements was greater than ±0.5 kg/cm. Note that the percentage of children requiring a third measurement was very low: 0.77% for height/length and 0.39% for weight. For the purposes of this study, the final length/height, and weight values were computed as the average of the first two measurements if the differences between them were less than ±0.5 kg/cm. In contrast, if the difference between them was greater, the third anthropometric measurement was considered, and the final value was the average between the two closest measurements. The aim of following this strategy was to minimize any possible measurement error due to the specific measurement procedure applied during the interview.

### 4.2. Data Variables

The key independent variable in this analysis was maternal employment (MLS). First, we measured it as a dichotomous variable that equals 1 for wage earning mothers and self-employed mothers working outside the household and 0 otherwise. Second, we measured it as a continuous variable using the average number of hours worked per day. Note that while the former was calculated using ENSANUT 2018, the latter was computed by means of ECV 2014. As maternal employment is measured contemporaneously, the estimated effects were limited by the influence of current household conditions on health and nutritional status. Note that we assumed zero hours worked for inactive mothers.

In our analysis, we used the local labor market conditions of women as our instrumental variables. The first instrument is measured as the cantonal average employment rate of women aged 15 and above, which is computed by dividing the number of employed and self-employed women by the total number of economically active women in the area, excluding unpaid self-employed women and those who work from home. This instrument can be computed using ECV 2014 and ENSANUT 2018. Meanwhile, the second instrument is measured as the mean daily hours worked by women aged 15 and above who are not unpaid self-employed, calculated by canton. This instrument is available in ECV 2014 only.

A large set of controls was used to explain the variability in the nutritional status of children. We included the following characteristics of children: (i) gender, (ii) age in months, and age squared, since the relationship between a child’s age and the dependent variable is non-linear. Information on the early initiation of breastfeeding, a key source of nutrition and immune protection for the newborn, is available only for children younger than 3 years old. Due to the high presence of missing information in this control, we excluded it from our analyses [50]. For mothers, we included: (iii) maternal age and age squared, based on the same rationale as for a child’s age, (iv) a mother’s height and weight, (v) maternal educational attainment level, (vi) marital status, and (vii) cultural origins (ethnicity). The latter is thought to play an important role in child nutrition since different ethnic groups in Ecuador may be associated with different levels of food intake, culture, and traditions. For the family and the environmental characteristics of children, the econometric specifications considered: (viii) household income in quintiles, a key determinant of the child health production function discussed in Section 2, (ix) paternal employment, (x) number of U5-year-old children in the household, (xi) area of residence (urban/rural), and (xii) number of women (aged 15 to 65) living in the same household, taken as a substitute for childcare when the mother works outside the home. Finally, we also included xiii) dwelling features such as overcrowding, inappropriate excreta disposal, and unsafe drinking water that might be relevant to the nutrition of children, i.e., our preferred specification. Table A1 describes all variables used in this study, while Table A2 in Appendix A shows the descriptive statistics of these variables. Note that survey weights were used for all mean calculations and standard errors were corrected for the clustering effect.

## 5. Empirical Results

### 5.1. Descriptive Statistics

**Figure 1:**
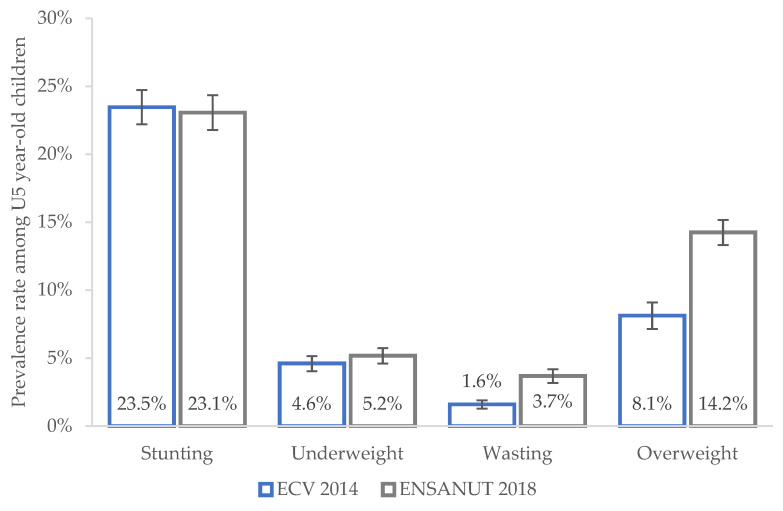
Nutritional status of children U5 years old. Notes: the figures presented here are estimates considering the sample of U5-year-old children living with their mothers and survey weights.

The data show that long-term, chronic malnutrition in Ecuador is a public health challenge of the first order compared to short-term malnutrition. For instance, according to ENSANUT 2018, the prevalence of stunting among U5-year-old children living with their mothers is as high as 23.1%, while wasting and underweight have a much lower prevalence of 3.7% and 5.2%, respectively. The percentage of overweight children is 14.2%, making it an important health issue in the country (see Figure 1). Similar (lower) rates for stunting and underweight (wasting and overweight) were reported by ECV (2014).

The socioeconomic characterization of the malnourished children is shown in Table 1. Note that boys have poorer nutritional status than girls, except for overweight, which has the same prevalence rate in both sexes. Further analysis showed that stunting was much greater among children of low educated mothers. Interestingly, the percentage of overweight children was much higher for mothers with a high level of education than for those with a low or intermediate level. As expected, the percentage of stunted children in poor households was higher than the percentage in richer households. A significant difference of 16.3 percentage points in the prevalence of stunting was found between the lowest and the highest family income quintiles. The phenomenon was the same for underweight children. Although not shown, the same SES gradient of child malnutrition was found when using the ECV 2014 dataset. The descriptive analysis reported in Table 1 was also calculated for ECV 2014. 

**Figure A1:**
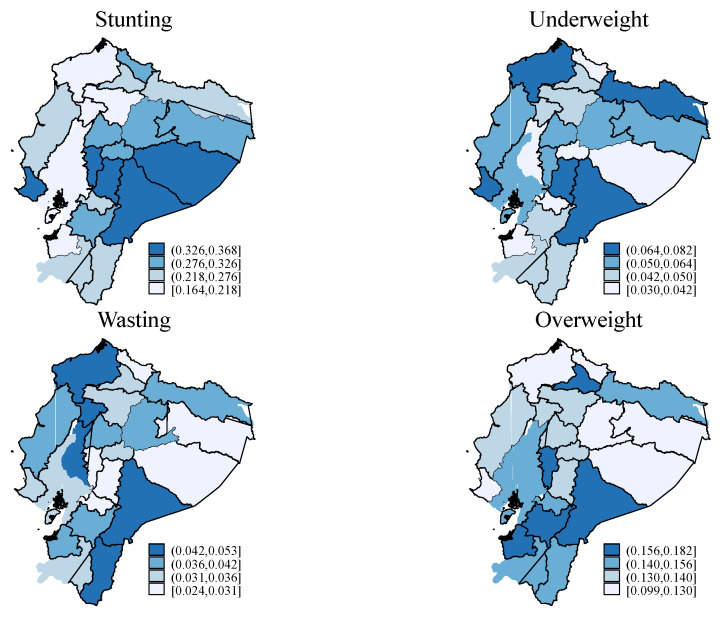
Prevalence of stunting, wasting, underweight and overweight by province, ENSANUT 2018.

**Figure A2:**
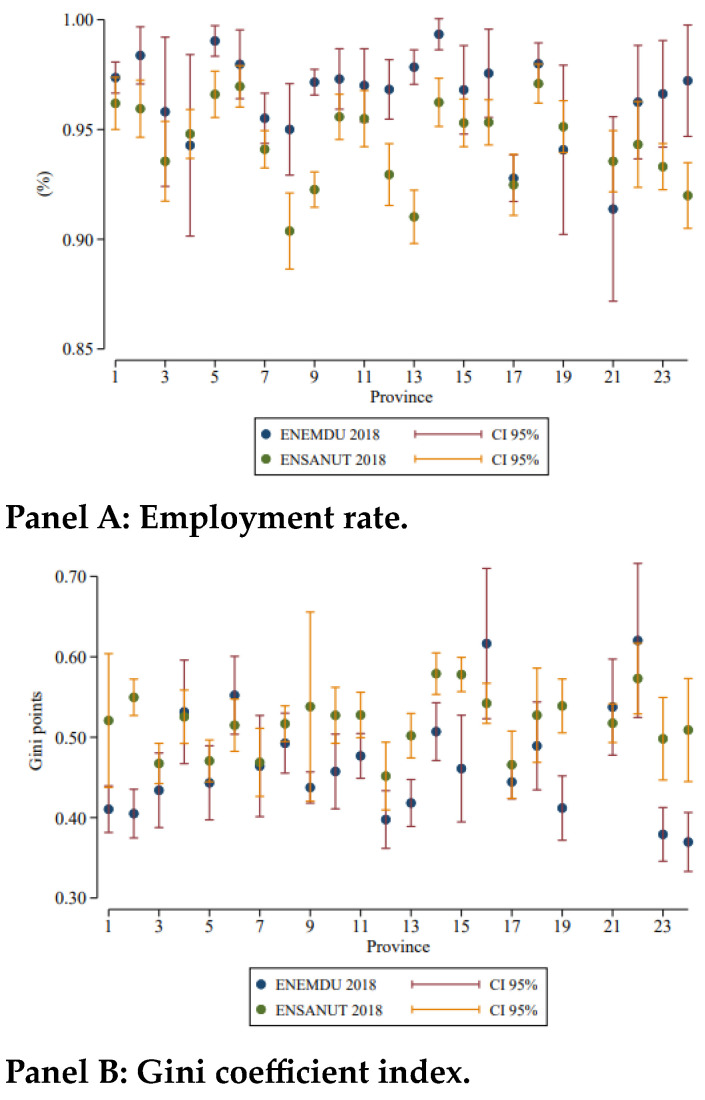

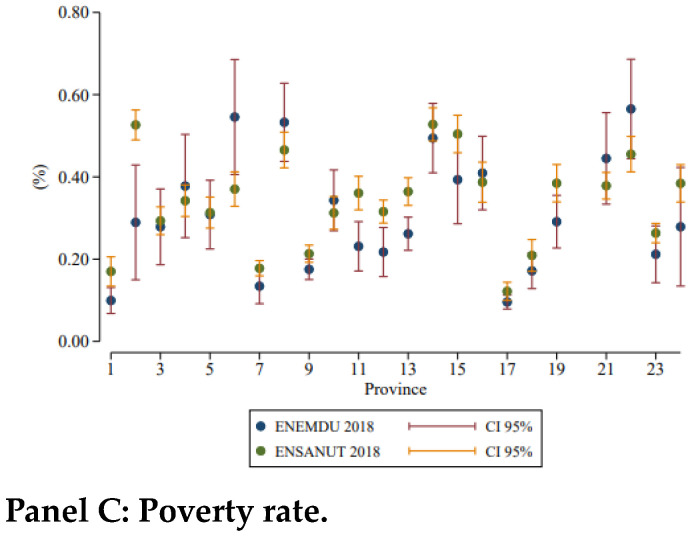
Provincial-level comparison of ENSANUT 2018 vs. ENEMDU 2018 covariates.

High levels of stunting were found mainly in the Amazon region and in the central highlands of Ecuador. However, the province that reported the highest percentage of stunting was in the coastal region. The south of the country was more affected by the prevalence of overweight, while the highest prevalence of wasting and underweight was mostly located in one province of the coastal region (Esmeraldas) and in the Amazon region (see Figure A1 in Appendix A).

Table 2 shows the structure of the condition of maternal labor activity in relation to the mothers’ socioeconomic background. Note that in Ecuador, the economically inactive group of mothers, regardless of place of residence, is larger than the economically active group (employed and unemployed). Interestingly, a considerable number of mothers in the no or primary and secondary education group were inactive, whereas mothers with tertiary education were more likely to be engaged in the labor force. For instance, 64.3% of total working-age mothers with tertiary education were employed, whereas 5.2% were unemployed.

Table 2 reveals that the share of employed (or unemployed) mothers rose or fell with the level of their family income. This result is unsurprising since mothers who work earn more money for their households. Finally, single and divorced mothers were more likely to be employed, while married women or women in couples were more likely to be inactive. A similar labor market composition was documented in the ECV 2014 dataset, although the largest group of mothers was that of employed mothers.

Overall, the descriptive evidence from both datasets indicated the existence of a social gradient of stunted children concentrated among socioeconomically vulnerable households, particularly those with low education levels and poor mothers. In contrast, overweight children were more likely to live in households with highly educated mothers. We did not find much variation in wasted children with respect to socioeconomic background. Moreover, most mothers were out of the labor market or inactive, especially those married or in a couple and with low education and a medium family income. In contrast, highly educated mothers with the highest family income were more likely to be engaged in the labor force as employed mothers.

### 5.2. OLS Estimates

The first step to empirically analyze the relationship between maternal labor supply and the nutritional status of children was to run an OLS model estimation (Table 3). While the baseline model considered up to four specifications, for reasons of space, we show only the estimates based on the widest set of controls. The estimates reported in Panel A indicate that dummy maternal employment is not significantly related to stunting in children. However, when MLS was proxied by the number of daily work hours (Panel B), the coefficient of maternal employment became, although modestly, positive and significantly related to stunting, i.e., children of working mothers were on average 0.4 percent more likely to be stunted than children whose mothers did not work (were unemployed or economically inactive) for each additional work hour.

Most of the coefficients of the controls were highly significant with the expected sign (see Table A3). For example, mothers with secondary and tertiary education were less likely to be associated with stunted children than mothers who had no or only primary education. Similarly, children of mothers living in richer households were less likely to be stunted than those who lived in poorer households. Interestingly, the number of U5 children residing in the household is positively related to the probability of having a stunted child. Moreover, a mother’s height and weight are associated with a lower probability of having a stunted child. Finally, Table A3 shows that membership in the indigenous ethnic group was more likely to be associated with child stunting than mothers with mestizo cultural origins. The coefficients associated with each control remain highly constant across specifications.

In contrast, the remaining columns of Table 3 indicate that maternal employment was unrelated to underweight, wasting, and overweight in children in Ecuador. The exception was the significant positive association between dummy maternal employment and overweight when controlling only for children’s characteristics.

In the next section, we test whether the relationship between maternal employment and nutritional status is exogenous.

### 5.3. Testing for Maternal Labor Supply Exogeneity

We used the Durbin–Wu–Hausman (DWH) test to evaluate the consistency of the OLS estimator of maternal employment compared to an alternative, consistently estimated IV model. We found that the DWH test rejected the null hypothesis of exogeneity in both surveys (F-statistic = 23.089, *p*-value = 0.000 in ENSANUT 2018; F-statistic = 57.191, *p*-value = 0.000 in ECV 2014). This suggests that maternal employment in the OLS framework for the equation of stunted children is endogenous. Thus, the estimates of maternal labor supply presented in Table 3 regarding the stunting model are inconsistent and biased.

However, the DWH tests did not reject the null hypothesis of exogeneity of maternal employment for the other outcomes. In the case of ENSANUT, the F-statistics were as follows: underweight 0.047 (*p*-value = 0.828), wasting 0.552 (*p*-value = 0.457), and overweight 0.724 (*p*-value = 0.395). For ECV 2014, the F-statistics were: underweight 0.173 (*p*-value = 0.279), wasting 5.968 (*p*-value = 0.015), and overweight 0.054 (*p*-value = 0.816). These results indicate that OLS provides consistent estimates of maternal employment for all nutritional statuses except stunting. Notwithstanding, we address the estimates of the causal effect of maternal labor supply on children’s nutrition based on the IV framework.

### 5.4. Instrumental Variable Estimates

In Table A4, we report the estimates of the first-stage regression based on the preferred specification. Using ENSANUT 2018, the findings indicate that the instrument’s mothers’ employment rate at the cantonal level is strongly correlated with maternal employment. Similarly, for ECV 2014, our estimates show that the average maternal hours worked at the cantonal level are significantly related to the endogenous maternal labor supply, although employment status is unrelated. Furthermore, at the bottom of this table, the Montiel-Olea and Pflueger robust effective first-stage F statistic and the corresponding *p*-value of the test of weak instruments are reported [51]. In both cases, this statistic was well above the critical values, and the null hypothesis of a weak instrument was rejected. Therefore, the relevance condition was satisfied.

Table 4 and Table A5 show the main findings of the paper. Table 4 displays the two IV-2SLS estimates for the outcome of stunting. Note that each structural form equation includes three cantonal-level variables (poverty, income inequality, and poor health status) to ensure the exogeneity of the instrument and, in this way, satisfy the validity condition. According to column (1), after controlling for observed characteristics (preferred specification), unobserved characteristics, and reverse causality, maternal employment status increases the probability of having a stunted U5-year-old child by roughly 18 percent compared to unemployed and inactive mothers. However, when we considered two instruments and ECV 2014, we found that the Wooldridge’s heteroscedasticity-robust score test rejected the null hypothesis of overidentifying restrictions at the 5% significance level (Chi2(1) = 7.758, *p*-value = 0.005). Hence, the estimates presented in column (2) are the exact identified model instrumenting the endogenous regressor with the mothers’ average number of hours worked at the cantonal level. Interestingly, we found that each additional hour of work per day spent by a working mother raised the probability of a stunted child by 4.2 percent with respect to unemployed and inactive mothers. Table 4 shows that most of the coefficients associated with the controls had the expected sign and were statistically significant. Based on ENSANUT 2018, we re-estimated the model, including preschool attendance as a further control. We found a roughly similar IV-2SLS-significant impact of maternal employment on child stunting (20.4 percent). However, as this information is available for only one child U5 year-old in the household, we excluded this regressor from the baseline estimates as its inclusion reduces the sample size (from N = 17,193 to N = 14.514 obs.).

In contrast, Table A5 shows that the effect of maternal employment on underweight, wasting, and overweight children in both surveys was again not significantly different from zero. The exception was the negative and significant, albeit very small, impact on wasting. Overall, these findings suggest that maternal employment in Ecuador might have an impact on stunting or chronic long-term malnutrition but does not affect these other malnutrition outcomes. Hence, the OLS model may, to a large extent, underestimate the effect of maternal labor supply on stunted children. We also re-estimated the models after excluding (i) the mother’s own labor supply and (ii) the mother`s own hours worked from the calculation of the instruments to avoid an in-built relationship. The drawback of this approach is the marked reduction in the number of observations, which weakens the reliability of the estimated instrument. Although not shown, estimates were qualitatively similar.

### 5.5. Heterogeneous Effects on Stunting in Children

In Table 5, we investigate some heterogeneous effects and explore whether and to what extent the impact of maternal employment on children’s stunting differs by age and a mother’s socioeconomic status. In panel A, we differentiate between children aged 0 and 2 and those aged between 3 and 5 years, corresponding to preschool-age children. The estimates report that the impact of maternal employment on children U5 appears to be quite homogenous, with no sizable effects by child age. This is in contrast with other evidence that points to heterogeneous effects when employing other health outcomes and child ages. In panel B, we present estimates once the sample is divided by family income quintiles. Interestingly, our findings show a stronger and statistically significant positive impact on stunting in children among the group of mothers living in high-income households, and this is true both when maternal employment is measured in terms of labor status or in hours worked. Similarly, the IV estimates of panel C performed on both surveys confirm a much larger and more significant impact on child stunting among mothers with tertiary education. These results are compatible with other findings indicating that the effect of maternal employment on overweight children is driven basically by those at the top of the income distribution and with more educated mothers [21,22,31].

Overall, and within the context of our data, this evidence would appear to suggest that more educated, richer mothers, hence those with higher skills and abilities, tend to produce a poorer nutritional status for their children when working outside the home. Indeed, according to Datar et al., the reduction in the consumption of healthy foods and the increase in that of unhealthy foods and sedentary behaviors, associated with higher maternal work, would appear to be stronger among high-SES groups [22].

Finally, given the evidence of transmission of body weight within the family through biological and shared environmental influences [52,53,54], we further explore whether the effect of maternal employment varies by a mother’s overweight status, i.e., a proxy of an obesogenic environment. We hypothesize that the positive influence of maternal employment on child stunting should be lower within households with a more favorable or less obesogenic environment acting as a protective factor. Panel D shows the corresponding IV estimates, and the results appear to be inconclusive. While the estimates for the ENSANUT 2018 dataset would lend some support to this assertion, as maternal employment among overweight and obese mothers (mother’s BMI ≥ 25) raises child stunting more, effects are just the opposite when using maternal working hours and the ECV 2014 sample. For both samples, a large Chi2 test statistic rejects the null of equality of the coefficient impact of maternal employment by overweight status when running an IV regression including an overweight (BMI ≥ 25) indicator and interaction terms with maternal employment and the remaining controls.

## 6. Robustness Checks

Table 6 explores the robustness of the main findings under two different approaches. The first was associated with using a different sample criterion, while the second considered the same sample as that of the baseline scenario but used a seemingly unrelated regression (SUR) bivariate probit model. Note that all these estimates were based on the preferred specification.

We report in columns (1) and (2) of Table 6 the OLS and IV-2SLS estimates, respectively, based on the ENSANUT 2018 survey, when the estimation sample was restricted to mothers engaged in the labor force only (economically active mothers). This resulted in a much smaller sample size. The instrument used in this framework was women’s employment rate at the cantonal level, considering economically active women as the reference population. Therefore, the analysis now focuses on the trade-off between maternal employment and unemployment. Interestingly, the results reported in column (1) are not statistically significant for any malnutrition outcome. Hence, they are similar to the OLS estimates based on the complete sample reported in Table 3. However, when the IV-2SLS model was run, we found a roughly similar effect of maternal employment on stunting in children. Working mothers appeared to have a 25.6 percent greater probability of having a stunted child than mothers who are unemployed, although this effect was significant at the 10% level.

The second robustness check considered the complete sample of working age mothers, or the baseline scenario, but ran a binary choice model with endogenous regressors. Specifically, a SUR bivariate probit model was fitted. This allowed for the two probit equations with correlated disturbance terms, which were assumed to come from a joint or bivariate normal distribution [35]. Column (3) of Table 6 reports the average partial effect of maternal employment on nutritional status. As expected, for stunted children, the coefficient was positive and highly significant. This suggests that maternal employment raises the probability of having a stunted child by 17.1 percent, i.e., this coefficient was similar in size to the impact found using the IV-2SLS model. Furthermore, we found that the error terms of both probit equations were correlated (ρ = −0.3562; S.E. 0.0652). A Wald test of ρ = 0 was rejected for the stunting equation (Chi2 = 24.6542, *p*-value = 0.0000), which reveals that maternal employment is endogenous. There may be unobservable characteristics of individuals that adversely influence stunted children and employed mothers, and the SUR bivariate probit model is recommended to obtain consistent estimates of the structural equation parameters. The estimates for the remaining children’s status variables were neither significant nor consistent. The null hypothesis of correlation between the error terms for the other outcomes was rejected at conventional levels. This is similar to the evidence shown by the DWH test.

### Impact of Maternal Labour Supply on Severe and Moderate Stunting

As a further robustness check, we present in Table 7 the causal effect of maternal employment on U5 children who suffer from moderate or severe stunting based on ENSANUT 2018. According to definitions based on consensus reached by child health experts [47], it is assumed moderate stunting: height/length for age Z-score in the range of −2 SD, −3 SD. Severe stunting: height/length for age Z-score in the range of −2 SD, −6 SD. Based on the preferred specification, we show that maternal employment appears to lead to an increase in the probability of having a moderately stunted child by 13.9 percent compared with maternal unemployment and inactivity. However, although the sign is as expected, we found a non-significant effect on the probability of having a severely stunted child.

## 7. Conclusions

Among the countries of South America, Ecuador has the highest percentage of stunting among U5-year-old children (roughly 23%), according to UNICEF [8]. Its neighbors, in contrast, are in much better nutritional condition. For instance, in 2015–2016, Colombia reported a stunting prevalence of 12.7%, while more recently, in 2019, Peru’s rate stood at 12.2% [8].

This study sought to determine whether maternal employment influences malnutrition in Ecuadorian U5-year-old children. Based on cross-sectional and individual-level data from ENSANUT 2018 and ECV 2014, we report evidence that, after accounting for the endogeneity of maternal employment, working mothers are from 4.2 to 18.1 percent more likely to have stunted or chronically malnourished children than unemployed and inactive mothers. Interestingly, we find no evidence of a differential impact of maternal employment by child age, although we do find larger impacts for children of mothers with high education and from more affluent families. However, our evidence is inconclusive regarding a mother’s overweight status. Overall, this result suggests that the additional income that a working mother may obtain (the income effect) does not compensate for the loss of time available for direct child care (the time constraint) [22]. These estimated effects of maternal employment on stunting in children are in agreement with those found in the literature for working mothers in low and lower-middle-income countries. For instance, Amaha and Woldeamanuel reported that unemployed mothers in Ethiopia were 23 percent (*p*-value < 0.01) less likely to have a stunted child than employed mothers [55]. Similarly, Rashad and Sharaf found that the probability of stunting among children of working mothers in Egypt is 18.6 percent higher (*p*-value < 0.05) [15]. Notwithstanding, these findings may have limited external validity in other socio-economic contexts.

When chronic malnutrition is disaggregated by severity, we find that maternal employment, once endogeneity has been accounted for, is also an equally remarkable determinant of a child’s risk of experiencing moderate stunting. That is, a working mother is roughly 14 percent more likely to have a moderately stunted child. Again, the existence of a socioeconomic gradient relative to child malnutrition is highly prevalent. Mothers in the poorest family income quintile and those with lower or no education seem to be at greater risk of raising a malnourished child. Interestingly, boys are at greater risk of malnutrition than girls. This seems to show that a child’s environment has an influence on whether they suffer detrimental effects on their health status due to malnutrition.

However, this paper finds no empirical evidence for the impact of maternal employment on children suffering from wasting, underweight, or overweight. The effects are negligible in both the OLS and IV-2SLS regression estimations. Our results are robust to an alternative statistical modeling approach and different samples of mothers.

Our estimates seem to support the hypothesis that only stunting is affected by maternal employment, as this nutritional status, which results from a prolonged exposure to insufficient nutrient intake and child care in early life (particularly during the first 1000 days of life since conception), appears to be more sensitive to maternal employment or the time spent by the mother working outside the home, leading to a greater impact on stunting compared to wasting, underweight, and overweight.

The results of this study highlight the importance of government interventions to tackle the adverse impact of maternal employment on child nutrition. Effective conditional cash transfers and/or in-kind family policies targeting poor and low-skilled mothers and designed to reduce the cost of raising children for vulnerable families are supported by our findings. These results should serve to stress the urgency of increasing the efficiency of government programs, such as Programa Aliméntate Ecuador, in the overall strategy of combating child malnutrition in Ecuador.

Overall, the findings of this paper reveal the existence of a socioeconomic gradient associated with malnourished children in Ecuador. That is, children whose mothers have lower socioeconomic status are more likely to suffer stunting, as documented elsewhere [56,57,58].

Potential selectivity bias due to the non-observation of U5-year-old children who did not survive until the date of data collection seems to be of no concern in our setting. Nevertheless, the percentage of mothers who reported dead U5-year-old children in the five years prior to the survey was less than 1% of the sample.

Our results should be interpreted with caution. For instance, the study focuses on how mothers’ labor market decisions impact their children’s nutrition in the short run. Any long-term effects are disregarded. In fact, maternal employment could have a positive net effect on child health in the long run. A deeper understanding of this effect is needed. Notably, the estimates documented here are based on a cross-sectional analysis. Consequently, some time-invariant unobserved characteristics may not be accounted for, despite the fact that we use an IV strategy. Finally, more research is needed to examine the merits and drawbacks of government policies in their efforts to reduce the adverse effects on children’s health of the trade-off described in this study.

## Figures and Tables

**Table 1:** Child malnutrition by socioeconomic characteristics. ENSANUT 2018.

Variable	Stunting	Underweight	Wasting	Overweight
Child’s sex				
Male	24.8%	6.0%	4.2%	14.2%
Female	21.2%	4.3%	3.1%	14.3%
Mothers’ education				
No education/primary	29.0%	6.4%	3.1%	13.3%
Secondary	22.7%	5.3%	4.1%	13.5%
Tertiary	17.1%	3.6%	3.1%	17.1%
Family income (in quintiles)				
Lowest	32.3%	7.1%	3.6%	15.2%
Second	23.9%	5.7%	3.7%	12.7%
Middle	22.6%	5.7%	3.5%	13.9%
Fourth	20.2%	5.1%	4.9%	13.6%
Highest	16.1%	2.2%	2.6%	15.7%
Source: ENSANUT 2018				

**Table 2:** Maternal activity by socioeconomic characteristics. ENSANUT 2018.

Variable	Employed	Unemployed	Inactive
Region of residence			
Urban	41.5%	3.4%	55.1%
Rural	44.6%	5.3%	50.2%
Mothers’ education			
No education/primary	38.6%	2.8%	58.6%
Secondary	37.5%	5.3%	57.2%
Tertiary	64.3%	5.2%	30.5%
Household income quintile			
Lowest	34.3%	7.5%	58.3%
Second	26.9%	5.6%	67.5%
Middle	30.8%	4.6%	64.6%
Fourth	50.5%	3.8%	45.7%
Highest	75.3%	2.0%	22.7%
Mothers’ marital status			
Married or union	40.8%	3.2%	55.9%
Divorced	56.6%	8.7%	34.7%
Single	52.2%	12.0%	35.7%
Source: ENSANUT 2018			

**Table 3:** OLS estimation: Impact of mothers’ employment on malnutrition among U5-year-old children.

Variables	Stunting	Underweight	Wasting	Overweight
Panel A				
ENSANUT 2018				
Maternal employment	0.001	−0.004	−0.000	0.006
	(0.007)	(0.004)	(0.003)	(0.006)
Observations	18,060	18,099	17,450	18,046
R-squared adjusted	0.0591	0.0109	0.00739	0.0151
Panel B				
ECV 2014				
Working hours	0.004 ***	−0.001	0.000	−0.000
	(0.001)	(0.001)	(0.000)	(0.001)
Observations	10,428	10,466	10,330	10,448
R-squared adjusted	0.134	0.0261	0.0106	0.0174

Notes: Standard errors in parentheses clustered by primary sampling units. *** *p* < 0.01, ** *p* < 0.05, * *p* < 0.1.

**Table 4:** IV–2SLS: Impact of maternal employment on stunting in children.

Variables	(1)	(2)
	ENSANUT 2018	ECV 2014
Maternal employment status	0.181 ***	
	(0.043)	
Maternal working hours		0.042 ***
		(0.006)
Child’s sex (male)	0.039 ***	0.035 ***
	(0.007)	(0.009)
Child’s age in months	0.004 ***	0.012 ***
	(0.001)	(0.001)
Child’s age in months squared	−0.000 ***	−0.000 ***
	(0.000)	(0.000)
Mother’s age	−0.011 **	−0.015 ***
	(0.004)	(0.005)
Mother’s age squared	0.000 **	0.000 **
	(0.000)	(0.000)
Mother’s height	−0.008 ***	−0.014 ***
	(0.001)	(0.001)
Mother’s weight	−0.002 ***	−0.002 ***
	(0.000)	(0.000)
Education (none or primary education)		
Secondary education	−0.042 ***	−0.035 ***
	(0.009)	(0.012)
Tertiary education	−0.098 ***	−0.061 ***
	(0.015)	(0.016)
Marital status (married or in union)		
Divorced	−0.039 **	−0.047
	(0.017)	(0.030)
Single	−0.001	−0.053 *
	(0.017)	(0.030)
Paternal employment	0.016	0.021
	(0.013)	(0.026)
Children in the household	0.038 ***	0.036 ***
	(0.008)	(0.009)
Women in the household	0.001	−0.010
	(0.005)	(0.007)
Area of residence (urban)	−0.015 *	0.000
	(0.009)	(0.013)
Overcrowding	0.020 **	0.019
	(0.009)	(0.012)
Inappropriate excreta disposal	0.028 ***	−0.021
	(0.009)	(0.014)
Safe water to drink	0.004	
	(0.007)	
Family income quintile (poorest)		
Second	0.013	−0.032 **
	(0.013)	(0.015)
Middle	−0.012	−0.074 ***
	(0.013)	(0.017)
Fourth	−0.002	−0.132 ***
	(0.013)	(0.020)
Highest	−0.012	−0.185 ***
	(0.012)	(0.026)
Cultural trace (mestizo)		
Indigenous	0.017	0.038 *
	(0.014)	(0.019)
Black	−0.022	0.005
	(0.015)	(0.020)
Montuvio	0.000	0.002
	(0.017)	(0.023)
White	−0.016	0.040
	(0.029)	(0.034)
Cluster poverty	0.088 ***	−0.030
	(0.033)	(0.034)
Cluster Gini index	−0.003	−0.010
	(0.047)	(0.099)
Cluster poor health status	−0.094	
	(0.064)	
Constant	1.585 ***	2.560 ***
	(0.115)	(0.162)
Observations	17,193	10,428
Test of weak instruments-*p*-value	0.000	0.000

Notes: Standard errors in parentheses clustered by primary sampling units; *** *p* < 0.01, ** *p* < 0.05, * *p* < 0.1. Safe drinking water is not available in ECV 2014.

**Table 5:** IV–2SLS: Impact of maternal employment on child stunting. Heterogenous effects by age, education, and family income.

Panel A	(1)		(2)
	Child age 0–2		Child age 3–5
Maternal employment status (ENSANUT 2018)	0.188 *** (0.058)		0.181 *** (0.058)
Maternal working hours (ECV 2014)	0.041 *** (0.008)		0.044 *** (0.008)
Observations ENSANUT (Obs. ECV)	10,096 (6068)		7097 (4360)
Panel B	(1)	(2)	(3)
	1st/2nd Quintiles	3rd Quintile	4th/5th Quintiles
Maternal employment status (ENSANUT 2018)	0.179 *** (0.059)	0.066 (0.080)	0.278 *** (0.080)
Maternal working hours (ECV 2014)	0.033 *** (0.009)	0.045 *** (0.010)	0.053 *** (0.010)
Observations ENSANUT (Obs. ECV)	7716 (4966)	3312 (2045)	6265 (3417)
Panel C	(1)		(2)
	Primary/No education		Secondary/Tertiary education
Maternal employment status (ENSANUT 2018)	0.172 *** (0.058)		0.180 *** (0.057)
Maternal working hours (ECV 2014)	0.031 *** (0.007)		0.055 *** (0.010)
Observations ENSANUT (Obs. ECV)	4594 (4212)		12,599 (6216)
Panel D	(1)		(2)
	Mother’s BMI < 25		Mother’s BMI ≥ 25
Maternal employment status (ENSANUT 2018)	0.131 * (0.067)		0.226 *** (0.053)
Maternal working hours (ECV 2014)	0.050 *** (0.009)		0.037 *** (0.007)
Observations ENSANUT (Obs. ECV)	6890 (4398)		10,303 (6030)

Notes: Regressions run based on the preferred specification, including cantonal level poverty and Gini indexes. Standard errors in parentheses clustered by primary sampling units; *** *p* < 0.01, ** *p* < 0.05, * *p* < 0.1.

**Table 6:** Impact of maternal employment on children’s malnutrition status. ENSANUT 2018.

Malnutrition Status	(1)	(2)	(3)
	Labor Force Sample		Working Age Sample
	OLS	IV-2SLS	Bivariate Probit
Stunting	0.019	0.256 *	0.171 ***
	(−0.016)	(0.150)	(0.0020)
Underweight	0.007	−0.011	−0.008
	(−0.008)	(0.076)	(0.008)
Wasting	0.005	−0.015	−0.010
	(−0.007)	(0.055)	(0.0006)
Overweight	0.009	0.103	0.028
	(−0.014)	(0.099)	(0.0015)

Notes: Regressions run based on the preferred specification, including cantonal-level poverty and Gini indexes. Standard errors have been clustered by primary sampling units. The coefficient obtained from the SUR bivariate probit model has been computed as the average partial effect. Standard errors for the average partial effects are computed using bootstrapping method with 100 repetitions. *** *p* < 0.01, ** *p* < 0.05, * *p* < 0.1.

**Table 7:** IV–2SLS: Impact of maternal employment on moderate and severe child stunting. ENSANUT 2018.

Variables	(1)	(2)
	Moderate	Severe
Maternal employment	0.139 ***	0.046
	(0.037)	(0.029)
Observations	17,194	17,194

Notes: Regressions run based on the preferred specification including cantonal level poverty and Gini indexes. Standard errors in parentheses clustered by primary sampling units; *** *p* < 0.01, ** *p* < 0.05, * *p* < 0.1.

## Data Availability

The datasets generated and/or analyzed during the current study are available on the INEC web page (http://www.ecuadorencifras.gob.ec/estadisticas). Accessed on 1 February 2021.

## Abbreviations

U5: under five years-old; IV: instrumental variable; OLS: ordinary least squares; 2SLS: two-stage least squares; US: United States; BMI: body mass index; MLS: mother labor supply; SUR: seemingly unrelated regression; ENSANUT: National Health and Nutrition Survey; ECV: Life Conditions Survey; PSU: primary sampling units; WHO: World Health Organization; SD: standard deviation; HAZ: height-for-age Z-score; WAZ: weight-for-age Z-score; WHZ: weight-for height Z-score; H: height; UNICEF: United Nations International Children’s Emergency Fund; DWH: Durbin-Wu-Hausman; SE: standard error.

## Appendix A

**Table A1:** Variable description.

Variable	Description
Stunting	Equals 1 if HAZ is lower than −2 SD; 0 otherwise.
Wasting	Equals 1 if WHZ is lower than −2 SD; 0 otherwise.
Underweight	Equals 1 if WAZ is lower than −2 SD; 0 otherwise.
Overweight	Equals 1 if WHZ is higher than +2 SD; 0 otherwise.
Maternal employment status	Equals 1 for wage earning mothers and self-employed mothers working outside the household; 0 otherwise.
Maternal hours worked	Average number of hours worked per day by the employed and self-employed mother.
Child’s sex	Equals 1 if child is male; 0 otherwise.
Child’s age in months	Age in months since birth.
Child’s age in months squared	Square of child’s age in months.
Mother’s age	Age in years since birth.
Mother’s age squared	Square of mother’s age in years.
Mother’s height	Height of the mother in centimeters.
Mother’s weight	Weight of the mother in kilograms.
Mother’s education	Dummies for educational attainment levels: Primary, secondary, and tertiary.
Marital status of the mother	Dummies for marital status of the mother: Married or in couple, divorced and single.
Paternal employment	Equals 1 for working fathers; 0 otherwise.
Children in the household	Number of all U5-year-old children living in the household.
Women in the household	Number of all women between 15 to 65 years old living in the same household as the children.
Area of residence	Equals 1 if area of residence is urban; 0 otherwise.
Overcrowding	Equals 1 if there are more than three people per bedroom exclusively for sleeping in the household; 0 otherwise.
Inappropriate excreta disposal	Equals 1 if the household does not have appropriate excreta disposal; 0 otherwise.
Safe drinking water	Equals 1 if the household has safe drinking water; 0 otherwise.
Family income quintile	Per capita household income in quintiles without the labor mother’s income.
Cultural origins	Dummies for the ethnicity of the child: Mestizo, indigenous, black, montuvio and white.
Cantonal-level poverty	Poverty headcount rate measured at cantonal level.
Cantonal-level Gini index	Gini index measured at cantonal level.
Cantonal-level health status	Percentage of poor health status (i.e., fair and bad health status) self-declared, per canton.

**Table A2:** Descriptive statistics. ENSANUT 2018.

Variable	Obs.	Mean	Std. Error	Min	Max
Child characteristics					
Stunting	18,921	0.231	0.006	0	1
Underweight	18,966	0.052	0.003	0	1
Wasting	18,286	0.037	0.003	0	1
Overweight	18,910	0.142	0.005	0	1
Sex (male)	20,204	0.514	0.006	0	1
Age in months	20,204	29.406	0.207	0	59
Mother’s characteristics					
Age	20,204	28.565	0.091	11	71
Height	19,117	154.147	0.108	110.50	199.00
Weight	19,117	64.318	0.012	29.50	160.00
Household’s characteristics					
Paternal employment	18,921	0.747	0.007	0	1
Children in the household	18,339	1.267	0.008	0	4
Women in the household	18,921	1.452	0.010	0	8
Area of residence (urban)	18,921	0.683	0.007	0	1
Overcrowding	18,921	0.254	0.007	0	1
Inappropriate excreta disposal	18,921	0.236	0.009	0	1
Safe water to drink	18,828	0.342	0.008	0	1
Activity condition					
Employed	20,204	0.435	0.007	0	1
Unemployed	20,204	0.047	0.003	0	1
Inactive	20,204	0.517	0.007	0	1
Under 15 years	20,204	0.001	0.000	0	1
Level of education					
No education/primary	20,204	0.244	0.006	0	1
Secondary	20,204	0.540	0.007	0	1
Tertiary	20,204	0.216	0.006	0	1
Marital status					
Married or union	20,201	0.798	0.006	0	1
Divorced	20,201	0.102	0.005	0	1
Single	20,201	0.100	0.004	0	1
Family income quintile					
Lowest	20,199	0.201	0.006	0	1
Second	20,199	0.204	0.006	0	1
Middle	20,199	0.196	0.005	0	1
Fourth	20,199	0.200	0.005	0	1
Highest	20,199	0.200	0.006	0	1
Cultural trace					
Indigenous	20,204	0.090	0.004	0	1
Black	20,204	0.042	0.003	0	1
Montuvio	20,204	0.051	0.003	0	1
Mestizo	20,204	0.803	0.006	0	1
White	20,204	0.014	0.002	0	1

**Table A3:** OLS estimation: the impact of mother’s employment on U5-year-old child nutritional status.

Variables	Stunting	Underweight	Wasting	Overweight
Maternal employment	0.001	−0.004	−0.000	0.006
	(0.007)	(0.004)	(0.003)	(0.006)
Child’s sex (male)	0.042 ***	0.012 ***	0.006 **	0.017 ***
	(0.006)	(0.003)	(0.003)	(0.005)
Child’s age in months	0.005 ***	−0.001 *	−0.003 ***	−0.007 ***
	(0.001)	(0.000)	(0.000)	(0.001)
Child’s age in months squared	−0.000 ***	0.000	0.000 ***	0.000 ***
	(0.000)	(0.000)	(0.000)	(0.000)
Mother’s age	−0.002	−0.001	−0.002	−0.000
	(0.004)	(0.002)	(0.002)	(0.003)
Mother’s age squared	0.000	0.000	0.000	0.000
	(0.000)	(0.000)	(0.000)	(0.000)
Mother’s height	−0.007 ***	−0.001 ***	0.001 **	0.001 **
	(0.001)	(0.000)	(0.000)	(0.000)
Mother’s weight	−0.002 ***	−0.001 ***	−0.000	0.001 ***
	(0.000)	(0.000)	(0.000)	(0.000)
Education (none or primary education)				
Secondary education	−0.037 ***	−0.007	0.002	0.003
	(0.009)	(0.005)	(0.004)	(0.007)
Tertiary education	−0.059 ***	−0.014 ***	0.003	0.017 *
	(0.011)	(0.005)	(0.005)	(0.009)
Marital status (married or in union)				
Divorced	−0.013	0.006	0.001	0.007
	(0.015)	(0.009)	(0.007)	(0.012)
Single	0.022	0.012	0.005	0.025 **
	(0.015)	(0.008)	(0.007)	(0.013)
Paternal employment	0.011	0.001	0.011 **	0.015
	(0.012)	(0.007)	(0.006)	(0.010)
Children in the household	0.031 ***	0.006	−0.005 *	0.003
	(0.007)	(0.004)	(0.003)	(0.006)
Women in the household	−0.002	−0.001	0.001	0.006
	(0.005)	(0.002)	(0.002)	(0.004)
Area of residence (urban)	−0.021 ***	−0.001	−0.004	−0.006
	(0.008)	(0.004)	(0.003)	(0.007)
Overcrowding	0.016 **	0.004	−0.001	−0.008
	(0.008)	(0.004)	(0.004)	(0.007)
Inappropriate excreta disposal	0.028 ***	0.015 ***	0.003	−0.007
	(0.008)	(0.004)	(0.003)	(0.007)
Safe drinking water	0.002	−0.001	−0.000	−0.007
	(0.007)	(0.003)	(0.003)	(0.006)
Family income quintile (poorest)				
Second	−0.017 *	−0.012 **	−0.002	0.019 **
	(0.010)	(0.006)	(0.005)	(0.008)
Middle	−0.045 ***	−0.006	−0.003	0.016 *
	(0.011)	(0.006)	(0.005)	(0.009)
Fourth	−0.026 **	−0.009	−0.010 **	−0.004
	(0.011)	(0.006)	(0.005)	(0.009)
Highest	−0.029 ***	−0.014 **	−0.007	0.018 *
	(0.011)	(0.006)	(0.005)	(0.010)
Cultural origins (mestizo)				
Indigenous	0.062 ***	−0.001	−0.005	0.026 ***
	(0.012)	(0.006)	(0.004)	(0.010)
Black	−0.030 **	0.020 **	0.010	−0.033**
	(0.015)	(0.010)	(0.008)	(0.013)
Montuvio	−0.032 **	−0.001	−0.006	−0.008
	(0.016)	(0.008)	(0.007)	(0.013)
White	−0.026	0.012	0.004	0.014
	(0.027)	(0.015)	(0.014)	(0.028)
Constant	1.501 ***	0.314 ***	0.018	−0.060
	(0.106)	(0.055)	(0.043)	(0.088)
Observations	18,060	18,099	17,450	18,046
R−squared adjusted	0.0591	0.0109	0.00739	0.0151

Notes: Standard errors in parentheses clustered by primary sampling units, *** *p* < 0.01, ** *p* < 0.05, * *p* < 0.1.

**Table A4:** First stage of the IV-2SLS: baseline specification.

Variables	Employment Status (ENSANUT 2018)	Daily Hours Worked ECV (2014)
Cantonal-level average employment status	0.913 ***	0.333
	(0.041)	(0.480)
Cantonal-level average daily working hours		0.703 ***
		(0.061)
Observations	17,193	10,428
Adj. R-squared	0.1960	0.2330
Partial R-squared	0.0358	0.0555
MOP Effective F statistic	484.366	155.893

Notes: Standard errors in parentheses clustered by primary sampling units. These estimations incorporate all the exogenous covariates including both cantonal-level poverty and Gini indexes. Safe drinking water is not available in ECV 2014. MOP stands for the Montiel-Olea & Pflueger (2013) robust first stage statistic. *** *p* < 0.01, ** *p* < 0.05, * *p* < 0.1.

**Table A5:** IV–2SLS: Impact of maternal employment on underweight, wasting and overweight status.

Variables	(1)	(2)	(3)	(4)	(5)	(6)
	ENSANUT 2018			ECV 2014		
	Underweight	Wasting	Overweight	Underweight	Wasting	Overweight
Maternal employment status	0.001	−0.012	0.032			
	(0.022)	(0.016)	(0.033)			
Maternal working hours				−0.004	−0.003 **	−0.001
				(0.003)	(0.002)	(0.003)
Constant	0.306 ***	0.033	−0.022	0.468 ***	0.044	0.009
	(0.057)	(0.045)	(0.091)	(0.084)	(0.044)	(0.083)
Observations	17,230	16,616	17,180	10,466	10,330	10,448

Notes: Standard errors in parentheses clustered by primary sampling units. These estimations incorporate all the exogenous covariates including both cantonal-level poverty and Gini indexes. Safe drinking water is not available in ECV 2014. *** *p* < 0.01, ** *p* < 0.05, * *p* < 0.1.
